# A deep learning based framework for the registration of three dimensional multi-modal medical images of the head

**DOI:** 10.1038/s41598-021-81044-7

**Published:** 2021-01-21

**Authors:** Kh Tohidul Islam, Sudanthi Wijewickrema, Stephen O’Leary

**Affiliations:** Department of Surgery (Otolaryngology), Faculty of Medicine, Dentistry and Health Sciences, University of Melbourne, Melbourne, VIC 3010 Australia

**Keywords:** Computer science, Software, Statistics

## Abstract

Image registration is a fundamental task in image analysis in which the transform that moves the coordinate system of one image to another is calculated. Registration of multi-modal medical images has important implications for clinical diagnosis, treatment planning, and image-guided surgery as it provides the means of bringing together complimentary information obtained from different image modalities. However, since different image modalities have different properties due to their different acquisition methods, it remains a challenging task to find a fast and accurate match between multi-modal images. Furthermore, due to reasons such as ethical issues and need for human expert intervention, it is difficult to collect a large database of labelled multi-modal medical images. In addition, manual input is required to determine the fixed and moving images as input to registration algorithms. In this paper, we address these issues and introduce a registration framework that (1) creates synthetic data to augment existing datasets, (2) generates ground truth data to be used in the training and testing of algorithms, (3) registers (using a combination of deep learning and conventional machine learning methods) multi-modal images in an accurate and fast manner, and (4) automatically classifies the image modality so that the process of registration can be fully automated. We validate the performance of the proposed framework on CT and MRI images of the head obtained from a publicly available registration database.

## Introduction

**Figure 1:**
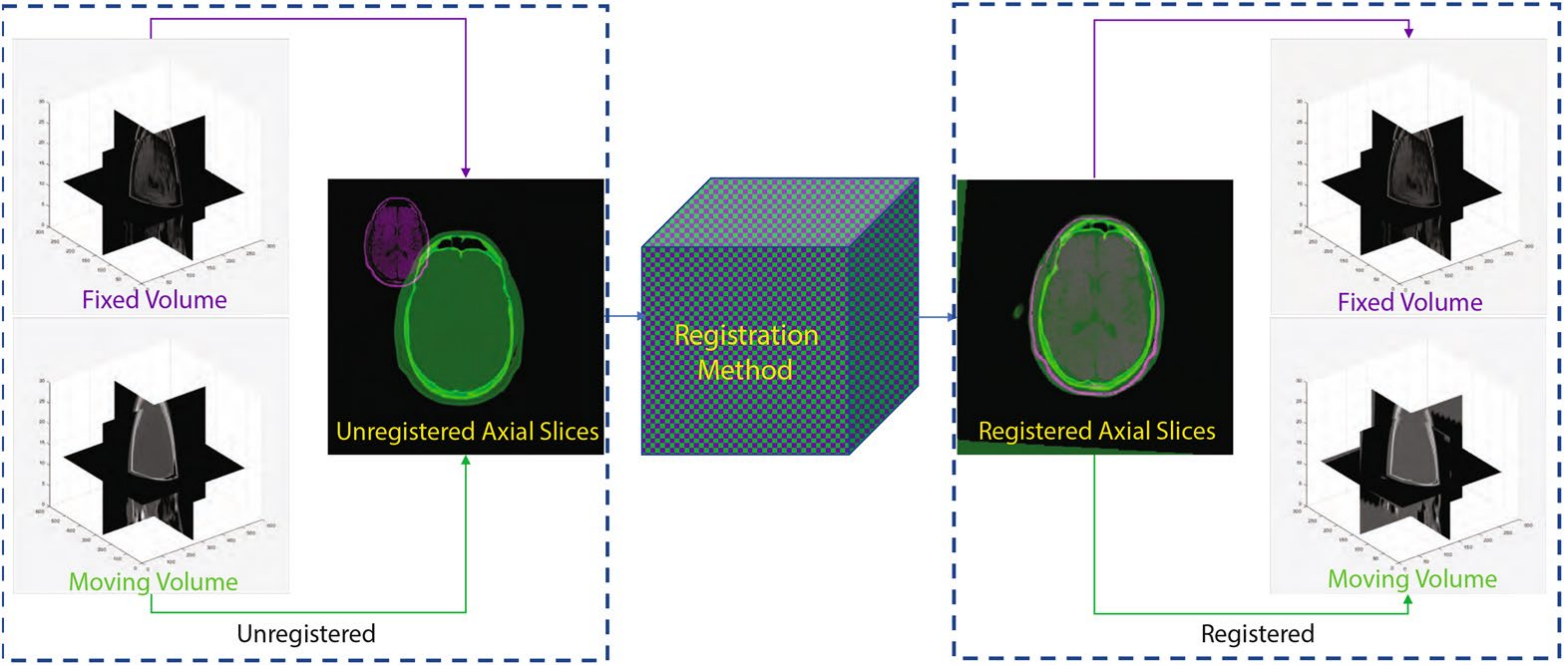
The process of 3D medical image registration.

Image registration is a spatial transformation process which brings different images into a single coordinate system. This enables direct comparison and integration of data obtained from multiple sources. Image registration has wide application in numerous fields, such as remote sensing, agriculture, infrared physics, and biomedical image analysis^1–3^. In the field of medical image analysis, it enables the integration of information from different temporal points and/or imaging modalities. For example, MRI (magnetic resonance imaging) scans of a patient at different times can show details of the growth of a tumor, comparison of pre- and post-operative scans can indicate the effects of a surgery, and using images from different imaging modalities (for example CT (computerized tomography), MRI (magnetic resonance imaging), and PET (positron emission tomography)) can provide additional information that can be used to improve the diagnosis of diseases. Figure 1 illustrates the process of 3D medical image registration.

Image registration methods can be broadly classified into intensity-based and feature-based methods depending on the matching technique used^4,5^. Assuming one of the images is fixed and the other is moving, both types of methods rely on determining the transform that minimises a cost function that defines the dissimilarity between the fixed image and the transformed moving image^6^. In intensity-based registration, the (dis)similarity of the two images is defined in terms of the correlation between pixel/voxel intensities. In contrast, in feature-based registration methods, image features (for example, edges, lines, contours, and point-based features) are extracted prior to the registration process and the correspondence between these features is used to define the (dis)similarity^5^. Feature-based image registration has the advantage of lower processing time, as only a smaller number of features are used in the (dis)similarity calculation when compared to each pixel (or voxel) used in intensity-based registration^7^.

Based on the nature of the transformation, registration methods can be classified into rigid-body and non-rigid registration. Rigid-body registration involves a combination of rotation and translation in order to bring the images into the same coordinate system^8^. However, if the images have geometric differences that cannot be accounted for by using rotation and translation, rigid-body registration may not be sufficient. In such situations non-rigid (non-linear, deformable, or elastic) registration is used^9^. Demons registration^10^, viscous fluid registration^11^, B-spline registration^12^, and finite element model (FEM) registration^13^ are some common methods used in non-rigid image registration. The anatomical differences present in inter-patient (between different patients) medical images necessitate the use of non-rigid registration^14^. In contrast, rigid-body registration has been found to be adequate for intra-patient image registration (where the images are of the same patient taken at different times and/or using different imaging techniques)^15,16^.

Over the last few decades, many methods have been introduced for medical image registration that typically use different cost functions and techniques. Mathematical optimisation as well as machine learning (both conventional methods and deep learning techniques) have been used for this purpose^17^. While the former is typically unsupervised, the latter can be either unsupervised or supervised. Optimization procedures minimise a distance function (or maximise a function that denotes similarity) between the fixed image and the moving image with respect to the registration (or transformation) parameters in an iterative process. Unsupervised machine learning techniques can also be used to optimise an objective function iteratively to find the registration parameters^18^. A limitation of these approaches is that the process has to be run from scratch for each new image pair. They are also computationally expensive and time-consuming^19,20^. In supervised machine learning, the model learns how a registration is performed through a training process involving pre-registered fixed and moving images. Although the training process could be resource intensive, once a model has been trained, the time taken for a registration is typically lower than that required for mathematical optimisation and unsupervised machine learning^21^.

When employing traditional Machine learning methods for feature-based registration, features are required to be extracted prior to the registration process. The decision of which features to be used in the registration typically depend on the application/user. In contrast, deep learning techniques are able to learn the ideal features for the registration directly from the input data as part of the training process^6^. As such, many deep learning techniques have been introduced for medical image registration tasks^22–24^.

Here, we explore the use of feature-based deep learning for 3D rigid-body registration of intra-patient, multi-modal (CT and MRI) images of the head. To this end, we first discuss a method of generating synthetic data to augment an existing registration dataset. Second, we describe a technique for ground truth generation for registration databases. Third, we introduce a registration method that incorporates the advantages of both conventional machine learning and deep learning. Fourth, we discuss a method to identify image modality in order to avoid manual intervention in selecting fixed and moving images. We validate the performance of our algorithms through experimental analyses on CT and MRI images of the head obtained from a publicly available medical image registration database.

## Related work

There are several ways in which deep learning has been employed in feature-based supervised registration of 3D multi-modal images. Predominantly, researchers have trained deep regression models to predict the registration parameters^18,25,26^. Deep learning has also been used as a method of pre-processing, for example, to determine control points, which were then used to determine the registration parameters^27^. Another method that has utilised deep learning for this purpose was to train a model to predict the image of one modality given that of another modality of the same individual and registering this image with the original image of the corresponding modality, thereby reducing the problem to a single mode registration^28,29^.

Chee and Wu^20^ designed a deep learning model called AIRNet (affine image registration network) to predict the affine transformation parameters to register two 3D images. A twelve-element vector (a flattened version of the affine transformation matrix) was used as the output. First, they used an encoder (adapted from 2D DenseNet)^30^ to extract features from the image pair. Then they concatenated these features and used them as input to several fully connected layers that performed the registration task in the form of regression. Other works, such as Chee and Wu^20^ and Kori and Krishnamurthi^31^ used a similar approach to predict affine transformation parameters by adapting a 2D VGG-19 network^32^.

Sloan et al.^33^ introduced a deep learning regression model to predict rigid-body transformation parameters of intra-patient T1 and T2 MRI images of the head. Initially, they trained their network using a series of synthetic mono-modal images, where the fixed and moving images were identical but for some changes in rotation and translation. Later on they re-trained their method to register multi-modal images. They used two different networks to predict the transformation parameters as two different pipelines using a CNN and a full convolution network (FCN). In these methods, they first extracted features from the fixed and moving images using the feature extraction layers of the networks and then concatenated these features before sending them through several convolutional and fully connected layers. They showed experimentally that their method performed well compared to other similar methods they considered. However, it remains to be seen how it performs when registering images of modalities with larger intensity differences such as MRI and CT.

Liu et al.^29^ proposed a synthetic image generation based approach using deep learning for multi-modal medical image registration. They established that if an input image of one modality could be predicted from that of another modality, and this predicted image is then registered with the input image of the same modality, the registration process can be simplified into a mono-modal one. To this end, they used a CNN with 10 convolutional layers combined with ReLU and batch normalization layers in order to learn the complex feature mapping of an image of the input modality to its corresponding output modality in an image-to-image regression approach^34^. They used T1, T2 and PD (proton density) weighted MRI images to train their CNN model by using the sum of squared differences (SSD) as the cost function. Once this image was generated, they performed a mono-modal registration on that and the original image of the same modality using mathematical optimization. Liu et al.^29^ compared their image generation method with other methods and stated that their method can produce more detailed synthetic images than others, which in turn can make the conventional registration task easier. Since their method was only tested on MRI images of different types, it is not clear how it will perform in registration between images of modalities with larger differences.

Zou et al.^27^ implemented interest/control point and feature extraction based deep learning models for rigid-body medical image registration. First, they used a FCN to perform a pixel-level interest/control point calculation. Then, they used a CNN for feature detection and matching. Next, they used the random sample consensus (RANSAC) algorithm to filter outliers and determined the transformation matrix with the most inliers by iteratively fitting transforms^35^. They compared their performance with a traditional feature-based registration method: scale-invariant feature transform (SIFT) for MRI and CT images and showed their method performed well^36^.

Miao et al.^26^ used a CNN regression model to predict rigid body transformations. Their model was trained on synthetic images generated using a manual transformation. They showed experimentally that their model outperformed traditional intensity-based registration methods with respect to accuracy and computational efficiency. Similarly, Zheng et al.^37^ also used a pre-trained VGG-type CNN architecture^32^ to perform multi-modal medical image registration. They used their CNN as a regression model to predict parameters for rigid-body transformations. Initially, their model was trained on a large number of synthetic images and then fine-tuned using a small number of image sets. Their model outperformed similar existing state-of-the-art methods in terms of both accuracy and computational efficiency.

## Proposed method

**Figure 2:**
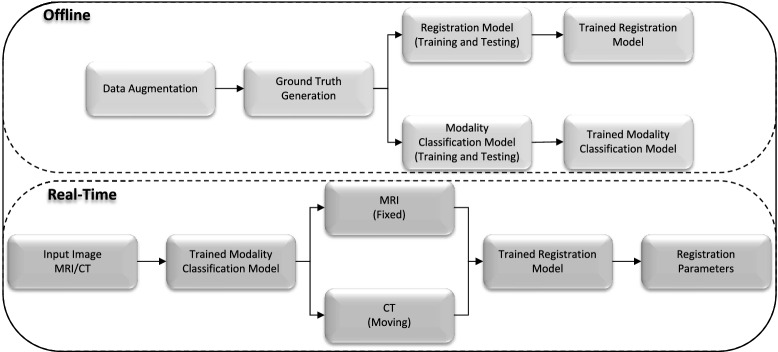
Overview of the proposed registration framework.

We consider four different aspects related to multi-modal 3D medical image registration and introduce methods for each. First, we discuss how to augment an existing registration database with synthetic images, as obtaining large databases for medical image registration is often impractical. Second, we address the problem of ground truth generation. In the absence of ground truth data generated by human experts, which may require considerable time commitments from them, researchers have used alternative methods such as using a validated algorithm^38,39^. Here, we introduce a method of increasing the accuracy of such validated algorithms when generating ground truth data for multi-modal medical image registration. Third, we introduce a registration method that comprises two steps: learning of image features best suited for the registration task (using deep learning) and determination of the registration parameters (using regression). Fourth, we discuss a method of identifying the image modality. This is an important task because the type of image determines whether it is used as the fixed or moving image, which is typically done manually. Identifying the image modality prior to registration enables the process to be fully automated. An overview of the proposed framework is shown in Fig. 2.

### Data augmentation

Data augmentation is a common practice in machine learning performed in order to increase the size of existing databases. It is typically done by generating synthetic data from the original data available in the database. The method used in generating synthetic images from the original dataset and the number of synthetic images to be generated are two important concerns in data augmentation. Many methods have been introduced in response to the former question, such as, random cropping^40^, mixing images^41^, generative adversarial networks^42^, neural style transfer^43^, and geometric transformations^44,45^. In our application, our focus is rigid body registration. As such, we generated synthetic images by rotating each image by a random angle (in the range of $[-15^0 \;\; 15^0]$) around a random axis and translating it by a random distance (in the range of $[-5 \;\; 5]$) along the coordinate axes. Not as much focus has been garnered by how many synthetic images should be generated for optimal training, and typically researchers have used arbitrary numbers that have performed well for their applications^40,45,46^. We employed an iterative training and testing procedure to identify the amount of synthetic data required to achieve best results. To this end, we generated an increasing number of synthetic data per original image (from 4 to 124 in steps of 5) and selected the number that provided best performance results.

### Generation of ground truth data

The accuracy of a model generated using supervised learning is largely dependent on the quality of the ground truth data used in its training. As such, ground truth generation is an essential part of any supervised learning problem. In medical image processing, this is typically done manually by human experts such as radiologists^47^. However, this is a time consuming, expensive, and cumbersome process. To avoid human involvement, some researchers have used validated algorithms to generate ground truth data^20,48^. In registration problems, this typically takes the form of mathematical optimisation^38^. However, one of the issues in mathematical optimisation in registration is that if the orientations of the two images are not reasonably similar, it is possible that the process would result in a local (and not global) minimum.

To reduce the impact of this issue, we introduce a method that realigns the fixed and moving images of the head so that their orientations are closer together at the start of the optimisation. To this end, we use a characteristic inherent to most medical images of the human body: symmetry^49^. As the human head is roughly ellipsoidal, principal component analysis (PCA)^50^ can be used as a simple, yet effective method of calculating symmetry. For images that have symmetry but are not ellipsoidal in nature, more complex symmetry calculation methods^51–53^ can be used.

**Figure 3:**
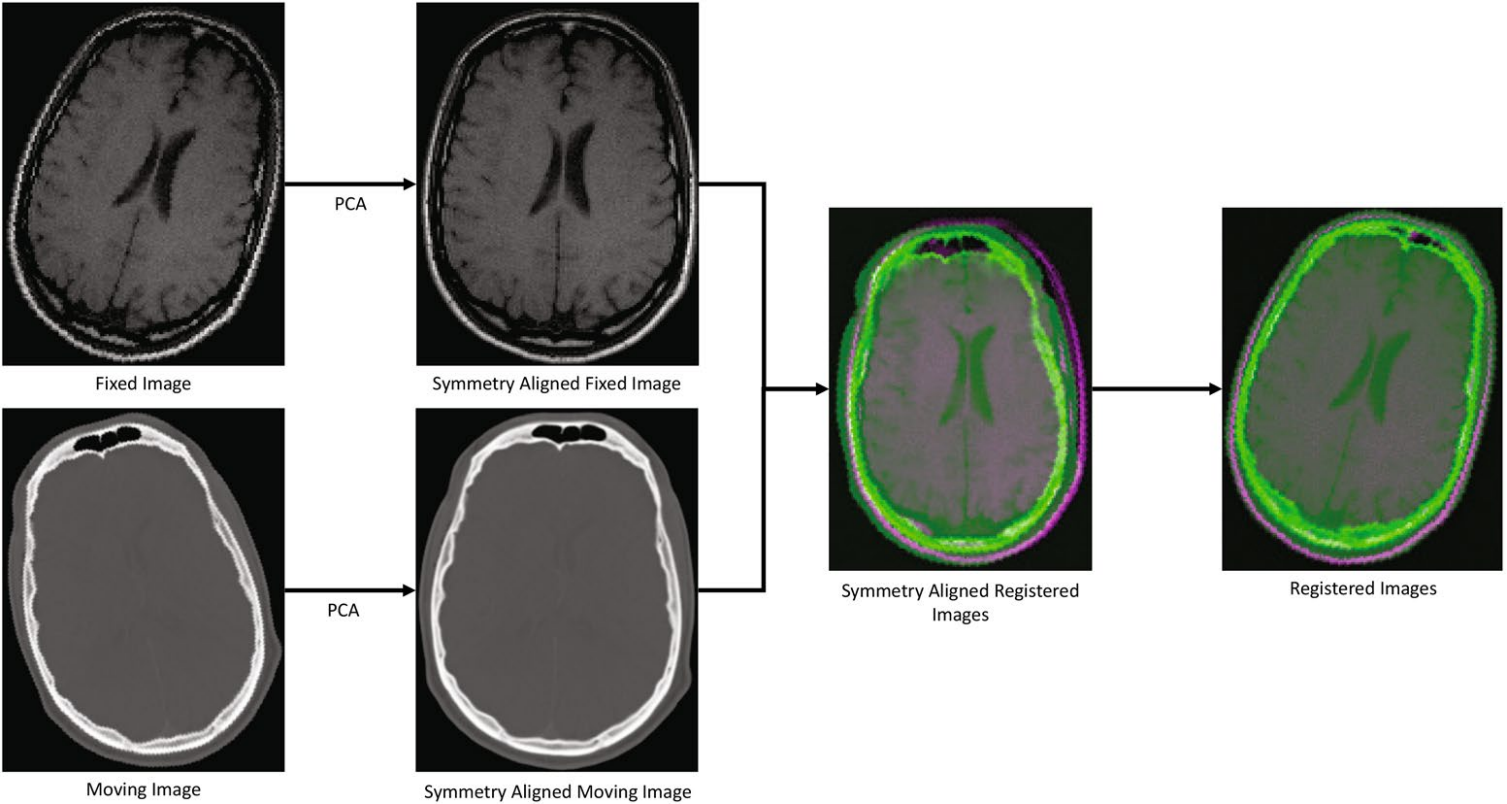
Ground truth generation process.

In order to calculate the PCA axes, we first resampled each image (fixed and moving) so that the scale of all three dimensions of the image were the same. This was done in order to preserve the real-world shape of the head. We then used Otsu’s two-level global thresholding^54^ method to remove the background points and extract a point cloud representing the head. We then calculated the principal components for the point cloud. Next, we reoriented the image so that its main axes were aligned along the principal components and the center of the image was at the mean location of the point cloud. This rigid body transformation can be represented by $$\left[ \begin{array}{cc} R &{} t \\ 0 &{} 1 \end{array} \right]$$, where *R* is the rotation matrix and *t* is the vector of translation parameters. Once the fixed and moving images were brought to their ‘symmetry’ orientations using the transformations $T_{Fix}$ and $T_{Mov}$ respectively, we registered these two images using One Plus One Evolutionary (intensity-based) optimisation^55^. In the optimisation process, we used a growth factor of $1.05\times 10^{00}$, epsilon of $1.5\times 10^{-06}$, initial radius of $6.25\times 10^{-03}$, maximum iterations of 100, number of spatial samples of 500, and number of histogram bins of 50. We considered all pixel values in the calculations. We then calculated the complete transformation that registered the original moving and fixed images as $T_{Comp} =T_{Fix}^{-1} T_{Reg} T_{Mov}$, where $T_{Reg}$ is the rigid body transformation resulting from registration of the symmetry aligned images. Figure 3 illustrates the process of ground truth generation.

### Registration of multi-modal images

**Figure 4:**
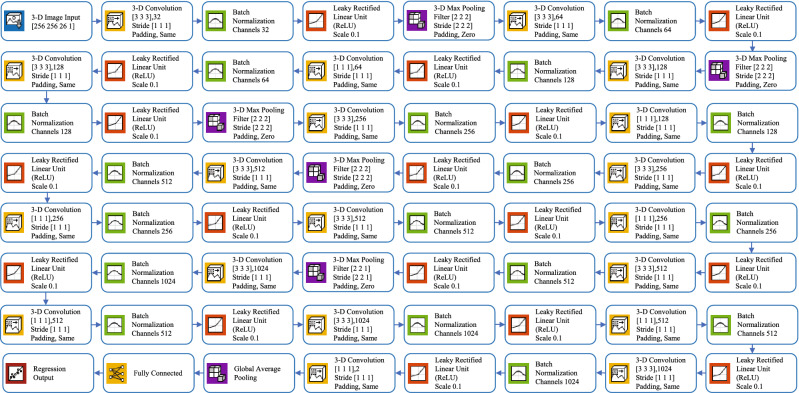
Architecture of the proposed 3D deep convolutional neural network.

We trained a CNN for the registration of multi-modal images and used it to extract the features best suited for our task. Then, we trained a regression model on these features to predict the registration parameters. The architecture of the CNN used here is shown in Fig. 4. The image input layer used was $256\times 256\times 26\times 1$. To speed up the training process and to reduce the sensitivity to network initialization, we used batch normalization layers after every convolution layer^56^. A Leaky Rectified Linear Unit (ReLU) layer was used after every batch normalization layer as a threshold operation to each element of the input, where any value less than zero was multiplied by a fixed scalar of 0.1^57^. Max pooling (3D) layers were used to perform down-sampling by dividing the 3D input into 3D pooling regions and computing the maximum of each region. A global average pooling layer was used before a fully connected layer to perform down-sampling by computing the mean of the height, width, and depth dimensions of the input and reduce the size of the activation without sacrificing performance. The final layer of the network was a regression output layer which computed the half-mean-squared-error loss. The output parameters were the three Euler angles representing the rotations around the three coordinate axes and the translations along the same. To train the network, we separated 30% of the data for validation and used stochastic gradient descent with a momentum (SGDM) optimizer of 0.9 and Max Epochs of 30^58^. We set the initial learning rate to 0.001 and dropped it to 0.0001 after 20 epochs.

**Figure 5:**
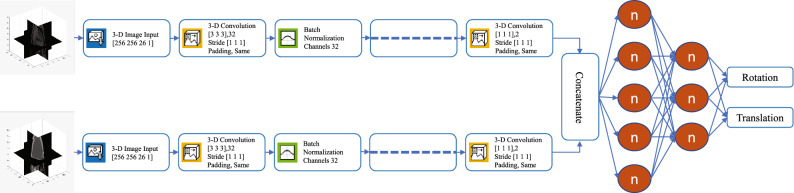
Complete architecture of the proposed method. For the full architecture of the DCNN, refer to Fig. 4.

Once the 3D DCNN was trained for registration, we used it to extract the features from the MRI and CT images. To this end, we activated the last convolution layer of the 3D DCNN (chosen using trial-and-error) which extracted 128 features for each image (see Fig. 4). We then concatenated the features of the MRI and CT images and used them as input to a regression Artificial Neural Network (ANN). The number of input neurons of the ANN was 256 (128 each for MRI and CT image features). This ANN had one hidden layer containing 10 neurons (chosen using trial-and-error) and the final output was connected to 6 output neurons representing the 6 transformation parameters which we aimed to predict. This ANN was a feedforward network with hyperbolic tangent sigmoid transfer functions in the hidden layer and linear transfer functions in the output layer^59^. We selected Bayesian regularization backpropagation^60^ as our training algorithm after comparing its performance with that of two others: Levenberg-Marquardt^61^ and scaled conjugate gradient^62^. The final architecture of the proposed method (3D DCNN for feature extraction and ANN for regression) is shown in Fig. 5.

### Identification of image modality

In order to make the proposed method fully automated, we developed a model to identify which of the input images was the fixed image and which was the moving image, and input them to the DCNN in the correct order. To this end, we used the same 3D DCNN architecture described above but replaced the fully connected layer with a softmax layer and the regression layer with a classification output layer. We used the same training strategy as above but changed the loss function to cross-entropy.

## Experimental results

### Experimental setup

All methods were implemented using the MATLAB academic framework, including the Deep Learning Toolbox and Image Processing Toolbox. A Hewlett-Packard Z6 G4 Workstation model computer powered by Intel Xeno Silver 4108 CPU (1.80 GHZ) with 16 GB of physical memory and 5 GB of graphics memory (NVIDIA QuADro P2000 GPU) was used for running the experiments. The operating system used was 64-bit Microsoft Windows 10 Education. Also, part of this work (feature extraction) was carried out on the Spartan High Performance Computing (HPC) system^63^.

We used the publicly available multi-modal 3D medical images provided by West et al.^64^ as part of The Retrospective Image Registration Evaluation (RIRE) dataset (Dataset can be downloaded from: http://www.insight-journal.org/rire/download_data.php) in our experiments. This dataset contains 3D multi-modal images which were collected at the same time. Here, we used T1 weighted MRI images from this dataset as the fixed images and the corresponding CT images as the moving images. First, we removed artefacts from the data manually as a pre-processing step. This was done because, unlike in manual ground truth generation where artefacts would be ignored by the human expert, in automatic methods they introduce a measure of error into the results. We then resized all images to $256\times 256\times 26$. Next, we split the dataset into training and test sets (60% and 40% respectively). As we used a freely available de-identified database, no ethics approval was required to carry out our experiments.

### Data augmentation

**Figure 6:**
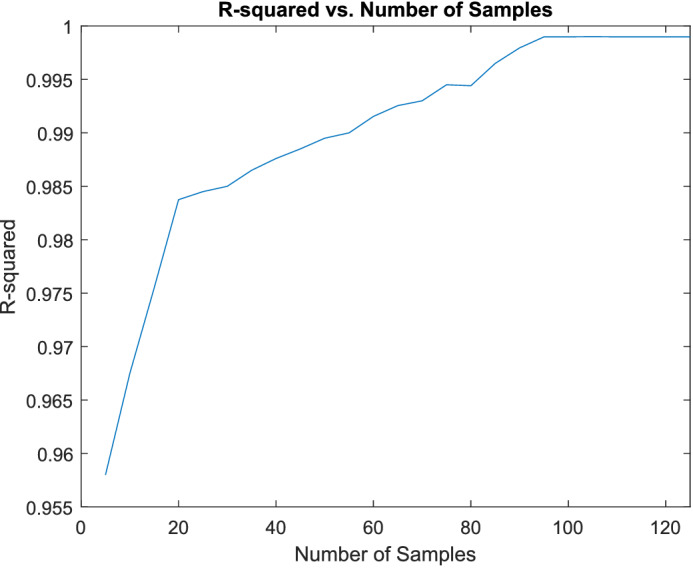
Change in network performance with the number of synthetic data.

As discussed above, we performed random rigid body transformations to generate different numbers of synthetic images in different steps ($n=4, \ldots ,124$ in steps of 5). To avoid bias, we allocated each augmented image to the same subset (training or test) as that of the original image it was generated from. We then trained and tested the DCNN regression model discussed above on the resulting datasets. To observe the performance of the regression models we generated regression plots for each step and calculated the $R^2$ value (which describes the proportion of the variance of the predicted parameters explained by the regression model). As can be seen from the results shown in Fig. 6, the accuracy of the model plateaued at around $n=94$. Thus, we chose $n=99$ as the number of synthetic images generated for each original image in the dataset.

### Generation of ground truth data

**Figure 7:**
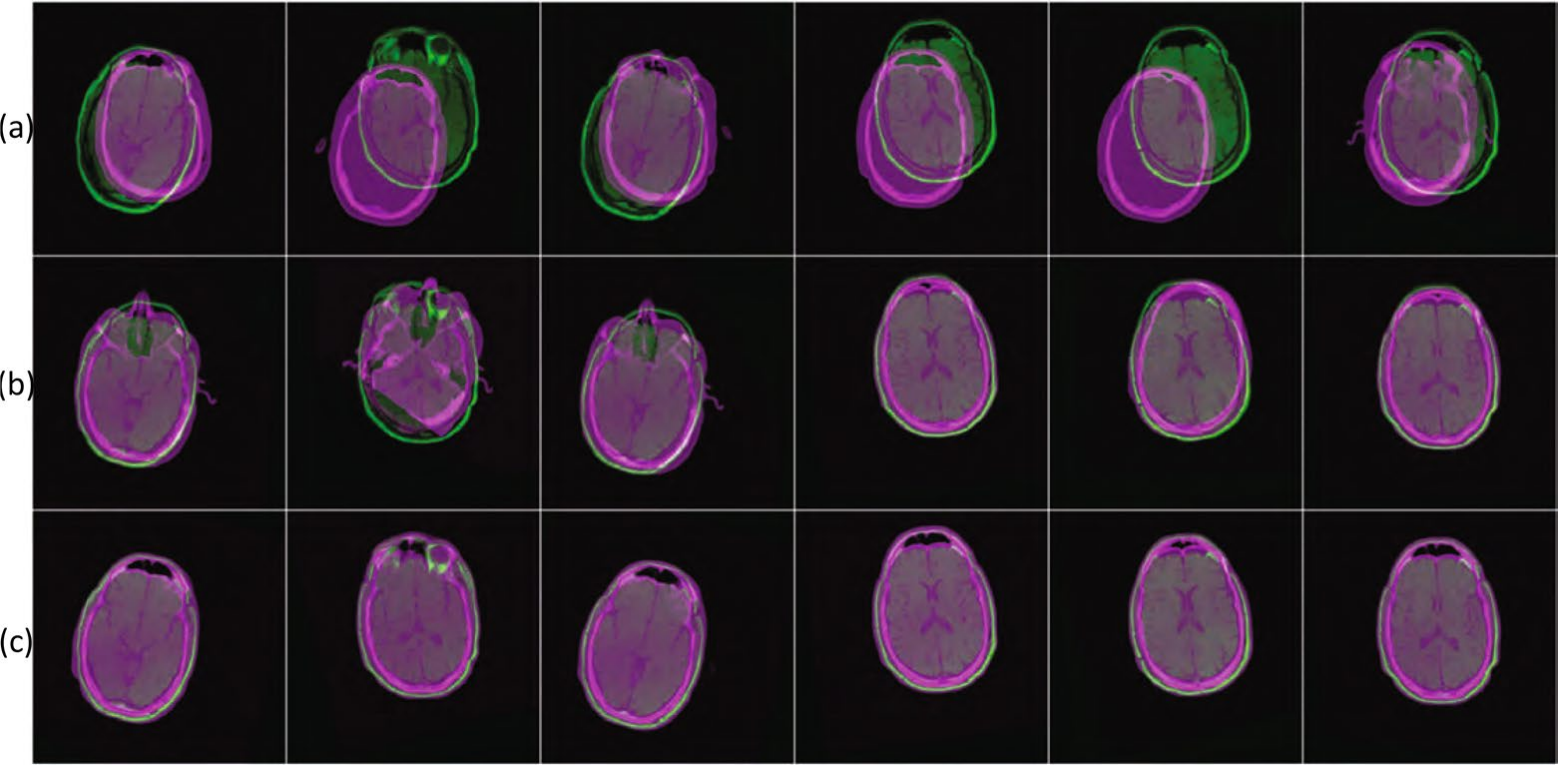
Example of registration without and with symmetry alignment. The rows show: (**a**) the fixed and moving images, (**b**) the results of the mathematical optimisation process without symmetry alignment, and (**c**) the results of the registration with symmetry alignment.

In the absence of manually obtained registration data, it is hard to determine the accuracy of the ground truth generation process using objective measures. Therefore, we conducted a manual assessment by a radiologist to evaluate the accuracy of the proposed ground truth generation process. To this end, we randomly selected 200 image pairs from the 1600 image pairs of our augmented dataset. We then displayed the axial, sagittal, and coronal slices of the registered image pairs obtained with and without the proposed modifications as methods A and B. The allocation of the method to A or B was randomised to remove bias. The assessor was asked to identify the best registration and given 3 options to choose from: A, B, or Both. The results of this assessment showed that 39.50% of the time, the 2 processes were similar, 28.00% of the time mathematical optimisation without symmetry alignment was better, and 32.50% of the time, mathematical optimisation with symmetry alignment was better. We show some visual results of this process in Fig. 7. Note from these results that in some cases (especially when the orientations of the fixed and moving images were close) the mathematical optimisation process performs well with or without symmetry alignment. However, in other instances (for example, where the orientations of the input images were vastly different), using symmetry alignment provided better registration results.

### Multi-modal image registration

#### Selection of regression model

**Figure 8:**
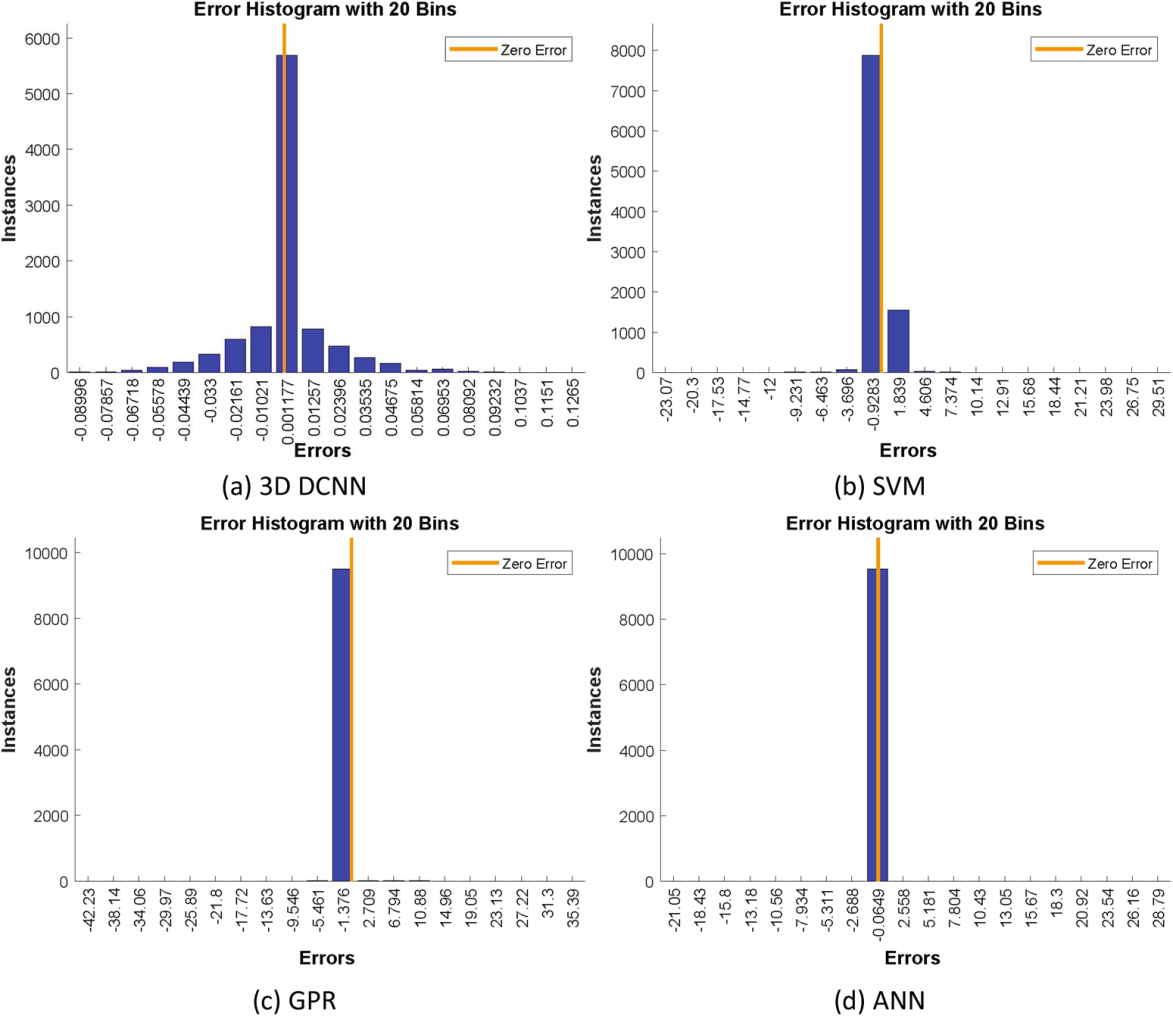
Histograms of errors for different regression models.

In order to select the best regression model for our application, we compared the performance of several models. First, we used the full 3D DCNN discussed above that was trained to predict registration parameters. Then, we used this DCNN as a feature extractor and combined it with several other regression models: Support vector machines (SVM), Gaussian process regression (GPR), and ANN. As SVM and GPR models were designed to predict one response at a time, we combined 6 prediction models to predict the 6 registration parameters. The resulting $R^2$ values for the regression models were: 0.958, 0.985, 0.990, and 0.998 for DCNN, SVM, GPR, and ANN respectively. We selected ANN as the proposed regression model as it gave the best $R^2$ value. Figure 8 shows the histograms of errors for the different regression models.

#### Complete registration process

We compared the performance of the proposed method with similar existing methods (Chee and Wu^20^, Zheng et al.^37^, Miao et al.^26^, Sloan et al.^33^, (CNN) Sloan et al.^33^, (FCN) Liu et al.^29^, and Zou et al.^27^) To this end, we generated the registration parameters from the trained networks, applied them to the moving image to get the registered image and then compared that to the ground truth (generated as discussed above). The metrics of comparison (for ground truth image *x* and predicted image *y*) were: Dice Similarity Coefficient (DSC), Jaccard Similarity Coefficient (JSC), Registration Precision ($R_p$), Registration Sensitivity ($R_s$), Contour Matching Score (CMS), and Structural Similarity Index Measure (SSIM)^65,66^. Equations 1 to 6 show the how these metrics were calculated. $\mu _{x}$ and $\mu _{y}$ represent the averages of the images, $\sigma ^2_{x}$ and $\sigma ^2_{y}$ are the variances, and $\sigma _{xy}$ is the cross-covariance. $C_{1}$ and $C_{2}$ are regularization constants for the luminance and contrast respectively. In addition, we considered the elapsed time ($E_{t(s)}$), the total processing time (in seconds), as a comparison metric.

1 $$\begin{aligned} DSC(\mathbf{x},\mathbf{y})&= \frac{2\left| \mathbf{x}\cap \mathbf{y}\right| }{\left| \mathbf{x}\right| +\left| \mathbf{y}\right| } \end{aligned}$$

2 $$\begin{aligned} JSC(\mathbf{x},\mathbf{y})&= \frac{\left| \mathbf{x}\cap \mathbf{y}\right| }{\left| \mathbf{x}\cup \mathbf{y}\right| } \end{aligned}$$

3 $$\begin{aligned} R_p(\mathbf{x},\mathbf{y})&= \frac{\left| \mathbf{x}\cap \mathbf{y}\right| }{\left| \mathbf{y} \right| } \end{aligned}$$

4 $$\begin{aligned} R_s(\mathbf{x},\mathbf{y})&= \frac{\left| \mathbf{x}\cap \mathbf{y}\right| }{\left| \mathbf{x} \right| } \end{aligned}$$

5 $$\begin{aligned} CMS(\mathbf{x},\mathbf{y})&= \frac{2 \times R_p(\mathbf{x},\mathbf{y}) \times R_s(\mathbf{x},\mathbf{y})}{R_p(\mathbf{x},\mathbf{y})+R_s(\mathbf{x},\mathbf{y})} \end{aligned}$$

6 $$\begin{aligned} SSIM(\mathbf{x},\mathbf{y})&= \frac{{(2\mu _{x}\mu _{y}+C_{1})(2\sigma _{xy} +C_{2})}}{(\mu _{x}^{2}+\mu _{y}^{2}+C_{1})(\sigma _{x}^{2}+\sigma _{y}^{2}+C_{2})} \end{aligned}$$

Table 1 shows the comparison of the proposed method with other existing methods on the augmented RIRE dataset. The results show the average performance on four different random training and testing subsets. As can be seen from the results, the proposed algorithm outperformed the other methods in all the metrics considered.

**Table 1:** MRI and CT images registration performance.

Methodology	DSC	JSC	$R_p$	$R_s$	CMS	SSIM	$E_{t(s)}$
Chee and Wu^20^	0.9825	0.9760	0.9790	0.9805	**0.9875**	0.9680	10.55
Zheng et al.^37^	0.9850	0.9625	0.9600	0.9810	0.9725	0.9610	8.30
Miao et al.^26^	0.9785	0.9625	0.9570	0.9685	0.9690	0.9520	40.80
Sloan et al.^33^ (CNN)	0.9780	**0.9765**	0.9760	0.9825	**0.9875**	0.9520	08.85
Sloan et al.^33^ (FCN)	**0.9885**	0.9645	0.9825	0.9860	0.9850	0.9650	09.55
Liu et al.^29^	0.9580	0.9350	0.9610	0.9780	0.9680	0.9365	43.85
Zou et al.^27^	0.9670	0.9560	0.9820	0.9855	0.9780	0.9485	14.10
**Proposed**	**0.9885**	**0.9765**	**0.9830**	**0.9870**	**0.9875**	**0.9685**	**02**.**80**

Best result for each metric is shown in bold.

**Figure 9:**
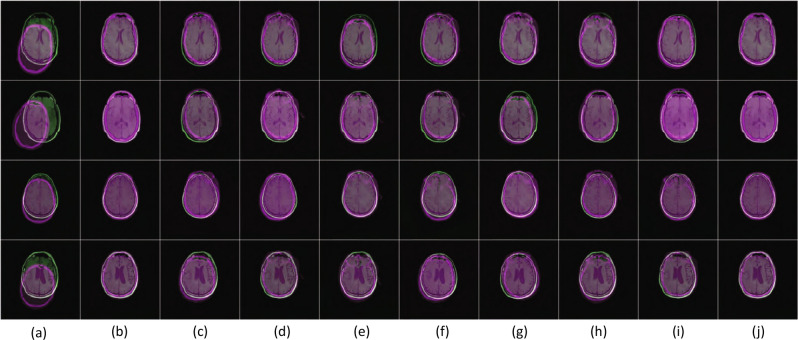
Examples of comparative performance of the registration methods. The columns show the axial views of: (**a**) input images, (**b**) ground truth, (**c**) Chee and Wu^20^, (**d**) Zheng et al.^37^, (**e**) Miao et al.^26^, (**f**) Sloan et al.^33^, (CNN) (**g**) Sloan et al.^33^, (FCN) (**h**) Liu et al.^29^, (**i**) Zou et al.^27^, and (**j**) the proposed method.

We further tested the proposed method against the performance of the above DNNs when used as feature extractors. To this end, we activated their last convolution layers, concatenated the features extracted from the MRI and CT images, and used this as the input to an ANN regression model (see section ‘Registration of Multi-Modal Images’ for details). Table 2 shows the results of this comparison. Registration results for some example image pairs are shown in Fig. 9.

**Table 2:** MRI and CT images registration performance (when the existing DNNs are used as feature extraction and coupled with a regression ANN).

Methodology	DSC	JSC	$R_p$	$R_s$	CMS	SSIM	$E_{t(s)}$
Chee and Wu^20^	0.9835	0.9780	0.9820	0.9815	0.9870	0.9685	9.50
Zheng et al.^37^	0.9865	0.9650	0.9630	0.9825	0.9730	0.9650	7.50
Miao et al.^26^	0.9790	0.9700	0.9610	0.9710	0.9730	0.9550	35.00
Sloan et al.^33^ (CNN)	0.9785	0.9780	0.9765	0.9850	0.9890	0.9530	07.35
Sloan et al.^33^ (FCN)	0.9895	0.9650	0.9835	0.9870	0.9855	0.9660	08.85
Liu et al.^29^	0.9610	0.9385	0.9670	0.9790	0.9700	0.9385	38.50
Zou et al.^27^	0.9680	0.9565	0.9835	0.9880	0.9785	0.9500	12.80
**Proposed**	**0.9910**	**0.9820**	**0.9903**	**0.9890**	**0.9930**	**0.9700**	**02**.**50**

Best result for each metric is shown in bold.

### Identification of image modality

To evaluate fixed and moving image classification performance, we used commonly utilized metrics (accuracy, sensitivity, specificity, precision, f-measure, and g-mean)^67–69^. These metrics are defined in Equations 7–12 with respect to values of the confusion matrix: true positives (*TP*), true negatives (*TN*), false positives (*FP*), and false negatives (*FN*) and results are shown in Table 3. We compared the performance of our method with some existing state-of-the-art pretrained architectures for image classification. The networks considered here were: AlexNet^70^, VGG-19^32^, SqueezeNet^71^, GoogLeNet^72^, and ResNet-101^73^. We modified the architectures of these networks so that they could accept 3D images as input.

7 $$\begin{aligned} Accuracy&= \frac{TP+TN}{TP+FN+FP+TN} \end{aligned}$$

8 $$\begin{aligned} Sensitivity&= \frac{TP}{TP+FN} \end{aligned}$$

9 $$\begin{aligned} Specificity&= \frac{TN}{TN+FP} \end{aligned}$$

10 $$\begin{aligned} Precision&= \frac{TP}{TP+FP} \end{aligned}$$

11 $$\begin{aligned} F{-}Measure&= 2\times \left( \frac{\frac{TP}{TP+FP} \times \frac{TP}{TP+FN}}{\frac{TP}{TP+FP}+\frac{TP}{TP+FN}}\right) \end{aligned}$$

12 $$\begin{aligned} G{-}Mean&= \sqrt{\frac{TP}{TP+FN}\times \frac{TN}{TN+FP}} \end{aligned}$$

**Table 3:** Performance comparison for the identification of image modality.

Architectures	Accuracy	Sensitivity	Specificity	Precision	F-measure	G-mean
AlexNet^70^	0.98520	0.97851	0.99500	0.99500	0.98625	0.98545
VGG-19^32^	0.98451	0.97632	0.99320	0.99530	0.98480	0.98785
SqueezeNet^71^	0.98475	0.97540	0.99885	0.99635	0.98645	0.98890
GoogLeNet^72^	0.99535	0.98965	0.99845	0.99845	0.99450	0.99480
ResNet-101^73^	0.99550	0.98975	0.99750	0.99750	0.99548	0.99565
**Proposed**	**0.99835**	**0.99565**	**0.99885**	**0.99885**	**0.99750**	**0.99750**

Best result for each metric is shown in bold.

## Conclusion

In this paper, we introduced a fully automated deep learning framework for 3D multi-modal medical image registration. To this end, we considered four aspects of image registration (data augmentation, ground truth generation, image registration, and identification of image modality) and introduced methods to address each. We validated the performance of the proposed methods on CT and MRI images of the head obtained from a publicly available database and showed that they outperformed similar existing methods. Although this framework was only tested on CT and MRI images of the head, it should be applicable to images of other organs and images of different modalities with minimal modifications (for example, more complex symmetry detection for data augmentation) and retraining.
